# Smarter Open Government Data for Society 5.0: Are Your Open Data Smart Enough?

**DOI:** 10.3390/s21155204

**Published:** 2021-07-31

**Authors:** Anastasija Nikiforova

**Affiliations:** “Innovative Information Technologies” Laboratory, Programming Department, Faculty of Computing, University of Latvia, Raina Boulevard 19, LV-1050 Riga, Latvia; anastasija.nikiforova@lu.lv

**Keywords:** Industry 4.0, Society 5.0, sensor, open data, open government data, OGD, OGD portal, real-time, COVID-19, sustainability, smart society, smart data, smart portal, timeliness, currentness, machine-redability

## Abstract

Nowadays, governments launch open government data (OGD) portals that provide data that can be accessed and used by everyone for their own needs. Although the potential economic value of open (government) data is assessed in millions and billions, not all open data are reused. Moreover, the open (government) data initiative as well as users’ intent for open (government) data are changing continuously and today, in line with IoT and smart city trends, real-time data and sensor-generated data have higher interest for users. These “smarter” open (government) data are also considered to be one of the crucial drivers for the sustainable economy, and might have an impact on information and communication technology (ICT) innovation and become a creativity bridge in developing a new ecosystem in Industry 4.0 and Society 5.0. The paper inspects OGD portals of 60 countries in order to understand the correspondence of their content to the Society 5.0 expectations. The paper provides a report on how much countries provide these data, focusing on some open (government) data success facilitating factors for both the portal in general and data sets of interest in particular. The presence of “smarter” data, their level of accessibility, availability, currency and timeliness, as well as support for users, are analyzed. The list of most competitive countries by data category are provided. This makes it possible to understand which OGD portals react to users’ needs, Industry 4.0 and Society 5.0 request the opening and updating of data for their further potential reuse, which is essential in the digital data-driven world.

## 1. Introduction

Today, more and more governments are launching open government data portals that provide data that can be accessed and used by everyone. This not only contributes to data-based decision-making, but also directly influences the trust, confidence and satisfaction of citizens with government, since open government data (OGD) allow citizens to monitor government performance and management and facilitates data-based solutions and services. The open (government) data ecosystem is very complex because it involves many stakeholders and creates a multi-directional channel where data can create the economic, social and environmental value [1,2,3,4], but the data themselves are significantly affected by political, social and economic outcomes, which affect both the data to be provided and their maintenance. Thus, there is a bi-causal relationship between the OGD and the economic and social aspects.

Today “openness” has become synonymous with “smartness” [5], which is also in line with the recent movement towards Smart Society, Smart Government supported by GovTech, and Smart Cities developed through Civic Tech, which have become dominant structures provided by information and communication technology (ICT) [6]. The common objective of both is to ensure a better and safer life for citizens through their engagement with government and technology. Therefore, the development of online public services is characterized by the use of collaborative methods involving different stakeholders, where open data play a key role. Governments are seen as the main beneficiaries of so-called GovTech, where policies on OGD and government transparency are being developed to enable citizens’ access to information and participation in government. However, Civic Tech includes a series of projects that use OGD to act in the public good [6]. This requires the opening of “right”/“correct” data, i.e., data interesting to both data re-users taking advantage of them and transforming them into solutions, and governments who facilitate the data opening process and benefit the consumer.

According to [2], the openness of data, both public and private, is not only one of the most crucial drivers for a sustainable economy, it can also impact on ICT [7,8] innovation and is seen as the creativity bridge in developing a new ecosystem in Industry 4.0 and Society 5.0. Open (government) data provide access to create added value in Industry 4.0 and Society 5.0, also known as the super-smart society, and support the idea of greater openness and accountability in business and administration governance [2,9,10]. Together, these concepts are seen as a driving force for new businesses and services that ensure the sustainable development of a human-centered society with open innovation [2,11] that resolves various social and economic challenges, incorporating innovations such as robots, big data and artificial intelligence (AI) [11], and is sometimes called the “society of imagination” [2,12]. Society 5.0 extends transparency and active participation in social issues by providing equal opportunities for all people, and by integrating innovative technologies and society. It is at the heart of the idea of open (government) data-equality and free access to data as a resource for their transformation into new services and solutions for both citizens, researchers, scientists, journalists and entrepreneurs/businesses. This became even more important when most countries were affected by pandemic, when data allow public agencies to predict and to alert citizens about potential risks. This was the case with *HealthMap AI*, which raised alarm even earlier than the research group *ProMed*, which noticed a post on social media talking about an “unknown pneumonia” [13], recognizing the threat on 30 December 2019.

However, pandemic-related open data is not the only topic of interest for society and businesses, in particular by pointing to the trend towards Smart cities, and the above mentioned Society 5.0 and Industry 4.0, thus creating more value. Today, the growing presence of sensors and IoT devices means that the physical world produces a huge amount of data of different types. Specific examples include data traffic management in ‘smart cities’ and ‘smart grids’ for automated electricity storage and distribution [14,15]. This trend has now reached open data and is considered to be an emerging trend [16] constituting the future of open data, as well as serving as a main facilitator of open data-based innovations and businesses of all sizes. However, although sensor data are found to be one of the most widely-used samples of data in citizen-related or so-called Citizen Science projects [17], only a very limited number of studies look at sensor-generated and/or real-time open (government) data despite their importance and potential value for both society and businesses. While some portals, such as Trentino, have been providing their users with sensor data already for 10 years [18], other countries still face challenges in this respect, even at the level of OGD portals.

The aim of this paper is to identify whether OGD portals in various countries support the open (government) data initiative and the movement to “smarter” open data, as well as provide their citizens with high-value data, and whether they are suited for further reuse. This would make it possible to understand which OGD portals can be used to obtain these data and allow less competitive portals to follow a learning-by-experience strategy. In addition to verifying whether this type of data is provided, their nature and type of access are covered*,* as well as such prerequisites for having the greatest impact as data timeliness and frequency of updates.

This paper is an extended version of the conference paper presented at the *Fourth International Conference on Multimedia Computing, Networking and Applications* [19]. The conference paper deals with pandemic-related OGD portals data answering the question: how quickly did data on COVID-19 (SARS-CoV) (as an example of a high value data set) come to open government data portals? This paper extends this study both at the level of detail and regarding the variety of topics considered. This paper focuses not only on pandemic-related data representing official statistics [20], but also on the second type of open urban government data, more precisely sensor and real-time data. This choice appears to be objective as the study [21] has proved that these open (government) data topics are currently crucial to society, e.g., collecting the highest number of tickets and proposals and the consequent initiatives and projects to be debated actively (in Madrid).

In regard to the research questions, there are primarily three:

RQ1: *What are the relationships between open (government) data, Society 5.0 and Industry 4.0?*

RQ2: *How do researchers assess the relevant topics identified as “smart” open (government) data {sensor-generated, real-time and COVID-19 data}? What is the state of the art for these topics? What lessons do these studies provide, i.e., the success stories and challenges of relevant data reuse?*

RQ3: *Do the OGD portals provide their users with real-time/sensor/COVID-19 data? Are these data suitable for reuse? Do OGD portals support the ideas of Society 5.0?*

The first two questions are answered as a result of a literature review, while the RQ3 is answered as a result of the inspection of the OGD portals of 60 countries, forming the core of the paper. To answer the RQ2 and RQ3, this study covers three categories/subsets of open (government) data, namely (a) COVID-19 data, (b) sensor data and (c) real-time data. These categories may overlap because medical data is one of the most popular types of sensor data, but sensor data in many cases are real-time data. COVID-19 OGD portals data waere covered in [19], so this paper will focus more on sensor and real-time OGD portals data. In terms of the RQ3 the following aspects are covered: (1) data presence or availability on the OGD portal and their findability, (2) their machine-readability, (3) their currency, i.e., are they regularly updated, (4) their accessibility through API that plays the most significant role for real-time and sensor data, (5) their popularity, if the portal allows these statistics, i.e., the number of views, downloads, reuses, rating, etc., (6) opportunity to provide a feedback, comment, suggestion or complaint and (7) their visibility for other users, (8) reuses/showcases based on data if the portal provides this information. The study covers data, impact, use and platform, where the last three aspects are considered as relatively rare, due to the complexity of evaluation, being also very time-consuming, and due to the immaturity of open (government) data practices [22]. However, these aspects affect open data usability and their value and allow the determination of factors that foster or hinder the increasing use of open data by society [2]. Both trends lead to the subject of the acceptance of new technology. This paper deals with both. The initial evaluation addressing the timeliness of COVID-19 data took place at the end of July 2020, and this extended analysis took place in May 2021 providing updated results.

In order to meet the aims of this research, the paper is organized in the following way: a brief overview of the main concepts and their value are presented in Section 2, the systematic literature review on the selected sub-types of “smarter” open (government) data is provided in Section 3, materials and methods are presented in Section 4, the methodology for the research is provided in Section 5, results and discussion are brought forth in Section 6, and the paper concludes in Section 7.

## 2. Background: Society 5.0 and Industry 4.0 in the Context of Open Government Data

The interconnection between topics of open (government) data, Society 5.0 and Industry 4.0 is a very new research direction in which the number of studies is currently very limited. One of the only studies that serve as an input for this study is [2], which was inspired by Zuiderwijk et al. [23]. In [23] “open data performance expectancy” is linked to reflection on the benefits of open data use, which has a positive impact on the behavioral intention to use and accept open data technologies. Before the relationship between these concepts is established, let us define our central concepts.

Society 5.0, also known as a super smart society, human-centered society and even a society of the imagination, refers to a society in which the imagination and creativity of different people lead to the solving of problems through new solutions and the creation of value, usually thanks to technological transformation stemming from Industry 4.0 [2,11].

Industry 4.0 has a number of different definitions, but most point to its relation to the digitization of processes, enabling the sustainable development of production, manufacturing, logistics, marketing, and sales. In general, it aims to promote sustainable development of the economic, environmental and social dimensions [2,11]. At the moment, the cyber-physical infrastructure is one of the most topical research areas, particularly for intelligent transportation, smart manufacturing, regional care, the smart food chain and medical treatment, and was developed based on the basis of IoT, sensors, advanced analytics, cloud computing, cyber security, smart and mobile applications, artificial intelligence and augmented reality [24].

Open (government) data, characterized as data that are available and accessible to everyone for their own needs, have huge potential. Recent evidence relates to the pandemic, when open (government) data were used to provide many solutions, ranging from simple visualization, such as interactive maps, to make the pandemic more actionable, to more specific and complicated services and solutions, including contact tracking applications, virus means of transmission, survival time on surfaces, potential antiviral treatments, as well as the extent to which individuals develop immunity after contracting the virus and healing [25]. The nature of depends on the data and the user, varying from epidemiological and health services to data on governmental measures, socioeconomic and environmental impacts.

According to Sołtysik-Piorunkiewicz et al. [2], there are similarities between products and services based on open (government) data and the concepts of Society 5.0 and Industry 4.0. These similarities are related to: (1) human-oriented action, (2) sustainable development, and (3) the physical-to-digital-to-physical loop, closely linked to geospatial data and real-time data together with the OGD portal, which, however, is capable of ensuring the sustainable development of a human-centered society. The co-creation of value for the sustainable ecosystem lies in the creation of a bridge between Industry 4.0 and Society 5.0, where the open (government) data play an important role as both a resource and, at the same time, as a tool. The authors point out that the main direction in presenting the benefits of open data products and services is to promote them as tools to provide real-time information on public issues, mainly in areas such as transport, education, culture and sport, economics and finance, and health. To this end, the authors have analyzed open data-based products and services developed in Europe between 2018 and 2020, covering regions in which they have been developed and the sectors to which they are related. Spain, the Netherlands and the UK, together with Germany, Italy, France, Belgium and Ireland, showing positive trends, produced the most open data-based solutions in 2018 and 2019. It is surprising that France has not been identified as a leader, given that its OGD portal has more than 2000 use-cases, which is the largest number of showcases among all portals analyzed in this paper.

One of the actions performed by the authors was the extraction of bigrams, where “real-time” was found to be the most popular bigram occurring in the description of solutions and services, based on open data, that are compatible with Society 5.0 and Industry 4.0. Other bigrams are related to specific business sectors dominated by transport, location, traffic and parking areas, which could be linked with the Internet of Things (IoT) and more precisely with the Internet of Transportation and Internet of Vehicles (IoV), i.e., smart transportation. They have also discovered a close relationship between these concepts and “sensors” closely associated with the topics above. This was also supported by going into more detail regarding specific keywords extracted, i.e., “air quality”, “weather”, “transport data”, “traffic”, etc., typically representing sensor-generated data. Although the transport sector was the largest business sector using the open data, this result now appears to be doubtful in terms of the pandemic, where health data would probably show better results compared to this study, since the research period was between January 2018 and January 2020, where it gained fifth place in popularity after “transport”, “education, culture & sport”, “economy & finance” and “environment”.

The keywords and bigrams identified lead the authors to conclude that open data are closely related to Society 5.0, sharing common concepts and techniques, including machine learning (ML), artificial intelligence (AI), predictive models, visualizations, interactive maps, etc., where the concept of AI in the context of open data and OGD in particular should follow both Industry 4.0 and Society 5.0 trends and be capable of producing more value from data [26]. While a typical open (government) data user may face challenges in applying AI to data, and rather will only do so in the near future, skilled developers are currently taking huge advantage of their use. Moreover, open data are now seen as an effective response to the industrial demands of AI [27,28] and able to contribute to (European) economy growth, AI development [27] and the overcoming of societal challenges [29,30]. AI is seen as a new support for decision-making. This is also in line with De Magalhães Santos [27], who has awakened the need for a new framework of regulation, ethics, inclusion, transparency and open governance for “political AI-based algorithms”. However, the use of AI in the context of open (government) data shows an even greater need for the data to meet machine-readability requirements and to respect the Berner-Lee 5-stars [26].

Another expressive solution demonstrating the potential of open (government) data moving toward Smart Society is the WeLive project [31], whose objective is to transform the current e-government approach by facilitating a more open model of design, production and delivery of public services, based on the collaboration among different stakeholders, i.e., citizens, public administrations, private companies and research institutes. The use of such concepts as open innovation, open data and open services paradigms allowed the authors to develop the framework, enabling the co-creation of urban apps. This led them to a novel ecosystem of tools built on open data, open services and open innovation paradigms and easily deployable in different public administrations, enabling the co-creation of urban apps based mainly on sensor- and open-data. The WeLive platform offers a set of tools that enables cooperation among different stakeholders, transforming needs into ideas, selecting the best ideas and creating the building blocks necessary for the desired solutions, composing them into mass market apps that are available through the WeLive Marketplace.

## 3. Literature Review

This section is devoted to RQ2. To understand the latest trends in studies on both selected open (government) data subtopics, namely (a) sensor data, (b) real-time data and (c) pandemic-related COVID-19 data, and understand whether they are really capable of creating value, a systematic review of scientific literature was conducted on digital libraries such as ACM Digital Library, ScienceDirect, Web of Science, IEEE Explore and Emerald Insight (Table 1). For the purpose of the study, the search was conducted querying these databases keywords: “*open data*”, “*open government data*” and “*OGD*” and depending on the subtopic (1) “*real-time*”, “*realtime*” and “*real time*”, (2) “*sensor*”, “*sensors*”, (3) “*COVID-19*”, “COVID”. They were combined using Boolean operators AND and OR.

First, a number of studies dealing with open (government) data as the primary object of the study were identified applying a query in the form of (“*open data*” OR “*open government data*” OR “*OGD*”) in the selected libraries. To find relevant primary articles dealing with open (government) data, rather than those which only mentioned the subject, the query was applied only to abstract and title. Then, how frequently the researchers referred to both open (government) data and an object under question—real-time data, sensor and COVID-19 data—where this object was the primary focus of open (government) data-related study, was examined. Only papers written in English were addressed. The period covered by these searches looked at the period 2013–2021 to gain insight into the popularity trends of these topics over the years and to select the latest studies to be analyzed in more detail.

The second search was conducted to find relevant open (government) data-related articles, dealing with artifacts under question, querying digital libraries in two ways: (1) abstract and title only to reveal studies where this topic is the primary focus, (2) anywhere in the text, i.e., including those which only mention the topic. Since the first option, i.e., primary studies, is of higher interest within the scope of this study, Table 2 shows the form of the query conducted in the digital library for studies related to “real-time” data.

The results of the analysis by digital library are summarized in Table 3, where:

- “total” stands for the total number of open (government) data-related studies returned as a result of querying the digital database for the relevant term to be met in the article—i.e., metadata or full-text, where the search was in the form of “*((“Abstract”:”open data” OR “Abstract”:”OGD” OR “Abstract”:”open government data”) OR (“Publication Title”:”open data” OR “Publication Title”:”OGD” OR “Publication Title”:”open government data”)) AND ((“Full Text & Metadata”:”(object under question version#1)”) OR (“Full Text & Metadata”:”(object under question version#1)*” (in the IEEE Xplore syntax, where “object under question version#n” is “real-time”, “sensor” or “COVID-19” with all respective versions listed above);
- “primary”—open (government) data-related studies, where the relevant search term is met in abstract and/or title. The respective queries for real-time related data are provided in Table 2;
- “ratio”—the ratio of primary studies to all open (government) data-related studies.

It was found that on average, 36% of open (government) data-related studies at least mention real-time open (government) data and 22% mention sensor-generated data, which corresponds to the increased popularity of IoT and Smart city movements. However, taking a step further and selecting “primary” studies, i.e., those that mention these search terms in the title or abstract, the results change significantly and only 0.44% address sensor-related issues and 6.93% real-time data-related topics.

It can therefore be concluded that, although many studies emphasize the significance of the concepts under consideration, real-time data and sensor-generated data, in particular, are more complementary materials that are rarely central to the study, despite their high importance and economic and innovative potential.

It is also important to point out that not all of these studies are relevant, i.e., mentioning the search terms in both the abstract and the title does not guarantee that the study covers the relevant aspects.

**Figure 1:**
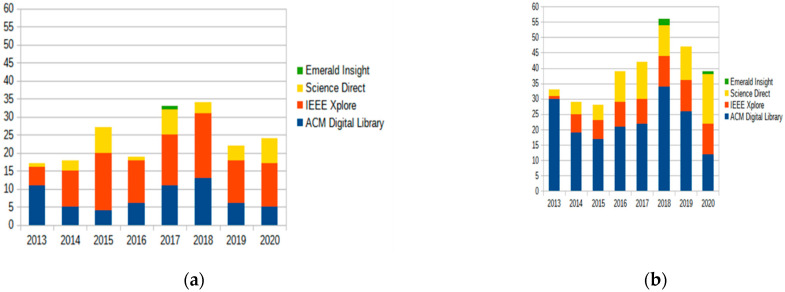
(**a**) Popularity of sensor open data-related studies by digital library between 2013 and 2020; (**b**) Popularity of real-time open data-related studies by digital library between 2013 and 2020.

Figure 1 provides more detailed insights, providing a summary not only for the digital library but also over the years. While IoT and Smart city are becoming more active topics from year to year, the study in question does not strongly support this fact, as the most “fruitful” year for both topics, sensor- and real-time data, was 2018, with a slight decline in their popularity in the scientific literature in the following years. However, despite the fact that the number of studies on both topics was the highest in 2018, the trend is positive and will increase in the coming years in all digital libraries inspected, with the exception of ACM Digital Library (also in line with [31]).

However, in order to understand whether these studies are actually relevant and determine whether these data are able to create value for both society and business, more in-depth analysis is needed. Thus, we cover some of the most interesting studies highlighting the potential of open (government) data, and sensor-, real-time- and COVID-19- open data in particular. They were selected by analyzing the list of studies selected during the previous stage, i.e., primary studies were inspected by analyzing their abstract, selecting the most relevant studies.

Yacchirema et al. [32] use a sensor-generated air pollution open data catalog, which is used in a system they propose focusing on the detecting of one of the most important sleep disorders, Obtrusive sleep apnea (OSA). They follow the treatment of this syndrome based on the use of open data processing, along with other factors such as sleep environment, sleep status, physical activities, and physiological parameters. The system obtains the open data (open data catalogs of smart city, more precisely the city of Valencia) on pollution levels and climatic conditions to advise elderly adults of the least polluted areas in the city where they can carry out physical activities without affecting their health. They highlight the competitiveness of their proposal compared to previous studies on this subject, related to the availability of open data available for re-use without the need to obtain the data, allowing for an extension of the overall perspective compared to previous studies focusing mainly on monitoring of physiological parameters.

Wang et al. [33,34] deal with the smart home topic where most devices access the Internet through a home gateway. They argue that most of the existing traffic classification solutions, such as deep packet inspection, cannot provide real-time application awareness for encrypted data traffic due to the unavailability of such data. Therefore, previous models proposed were suitable for unencrypted data. Today, however, when such data are available in the form of open data to everyone (200,000 samples of encrypted data obtained from 15 applications in this particular case), the authors were able to propose a software-defined network home gateway (SDN-HGW) framework to manage distributed smart home networks and support the SDN controller of the core network, where the SDN controller enables efficient network quality-of-service management based on real-time traffic monitoring and resource allocation of the core network for both types of data flows, encrypted or unencrypted.

According to Simonetti et al. [35], the relatively high global acquisition frequency and open data policy allow land cover mapping and monitoring in nearly real-time, using automated tools such as the phenology-based synthesis (PBS) classifier. A fully automatic PBS classification algorithm based on medium spatial resolution satellite data was developed using the Google Earth Engine cloud computing platform. Seasonality of vegetation, particularly in tropical dry areas, may result in conventional algorithms based on a single-date image classification “misclassifying” land cover types, since the selected date can only reflect a certain stage of the natural phenological cycle. The use of open data allows the PBS classifier to operate with the occurrence rules applied to the selection of single date image classifications within the study area, in order to assign the most appropriate land cover class. This opportunity led the authors to an overall accuracy of over 90%, thereby demonstrating the potential of the classifier and the power of cloud computing in geospatial sciences.

A similar observation by Dell’Acqua et al. [36] is that the recent trends in open Earth observation (EO) data have restored a general interest in satellite-based monitoring and mapping of the Earth’s surface. The open policy, now applied to LANDSAT data, and the launch of Sentinel operations, whose data are freely distributed even for commercial purposes, has eliminated a financial barrier to the wider use of Earth observation data in business activities, especially those with a narrow financial margin.

Shimauchi et al. [37] refer to a health-care topic, more precisely by proposing a semi-real-time RRI (R-R interval) estimation of wearable electrocardiogram (ECG) devices, which are widely used in various personal healthcare-based applications based on heart rate variability (HRV). The authors note that this had previously been achieved mainly by visual testing by medical experts, thus containing noise and artifacts in the data. Therefore, they propose a solution based on pseudo-ECG data which they have extracted from open data recorded with shirt-type wearable ECG devices, and actual ECG data recorded by a shirt-wearable ECG device during an exercise activity. Furthermore, their results show that pseudo data, i.e., the open data used, are almost as accurate as actual data retrieved from devices within the scope of the experiment, thereby encouraging others to use these in their studies.

Chen et al. [38] present a participatory urban-sensing framework for fine particulate matters PM2.5, which, among all pollutants, are directly related to a variety of serious health concerns, such as lung cancer, premature death, asthma, and cardiovascular and respiratory diseases. The use of real-time open data allows them to monitor this with more than 2500 devices deployed in Taiwan and 29 other countries, which is one of the largest deployment projects for PM2.5 monitoring in the world. The data they collect are released in real time and in an open data manner, and are available to all academic organizations and research groups. This allows researchers to collect data and conduct research on air pollution along with any associated respiratory diseases. This has contributed to the development of other products and services using data which has been made open, thereby creating a chain of valuable open data-based solutions and services.

Stieb et al. [39] studied the relationship between COVID-19 and PM2.5, where the data on COVID-19 was obtained from a Canadian open data portal. The combination of two sources and types of data, after monitoring for provinces, temperature, demographic and health characteristics and days since the maximum incidence in each health region, showed a positive relationship between long-term PM2.5 exposure and the incidence of COVID-19.

Another study on this topic is [40], which provides a simple approach to the cheaper estimation of PM2.5 concentration. The proposed approach is based on image processing schemes and a simple linear regression model, using high and low PM2.5 concentration images to obtain the difference between them, which is then used to find the regions with the greatest impact. The development of the approach is closely linked to open data, more precisely open data on the hourly PM2.5 concentration and relative humidity, used as input data for testing the model, which allowed a significant reduction in resources spent on its development and testing.

COVID-19 OGD was also used by López et al. [41], proposing a SARS-CoV-2 virus transmission model based on human flow networks, where the daily activities of citizens are closely linked to the evolution of the pandemic and the corresponding restrictions applied to individual countries and regions. This study added new perspectives for the evolution of studies of infected people between states and allows the modeling of different scenarios, as well as illustrating the evolution of and trends in the pandemic, based on open OGD updated by governments on a daily basis, thus contributing to the fight against the pandemic.

The study of Tan et al. [42] is related to another sensitive issue—dementia, whose early detection is essential for older people to extend their independent life span, and whose early symptoms are noticeable in everyday activities, such as front-door events. Therefore, the authors present a classification scheme for front-door events (exit, enter, visitor, other, and brief-return-and-exit (BRE)), validated using open data sets collected during another long-term real-life experiment in a smart house using non-invasive wireless binary sensors, i.e., another study with another aim. The use of open data allowed the authors to propose and train an algorithm that could be a useful tool for the detection of forgetting events and, as a result, to detect symptoms of dementia.

Aziz et al. [43] point out that the recent increase in open data analytics and data integration technologies provides new opportunities to optimize the operation of highway infrastructure, such as the use of cameras and sensors to monitor real-time traffic flows, speeds and travel times, GPS data for driving and monitoring journey times, data from telecommunications operators/social media applications to improve awareness of journey patterns, data on condition of roads, etc. Opening data on infrastructure and network management and linking these to data from different sources provides new optimization capabilities, such as road asset maintenance, cross-asset comparisons, better information for road users, etc.

Another study carried out by Stone et al. [44] estimated the benefits that public transport authorities could gain from the opening and publishing of data in an efficient way, by presenting the use of transport for London, and proving both qualitatively and quantitatively that the benefits of data opening can be as high as those of major transport infrastructure development projects. The main finding is that achieving an open data approach in public transport helps to deliver a clear commitment that data belong to the public and that third parties should be allowed to use and re-use data, by having a strong digital strategy, and through creating a strong partnerships with data management organizations that can support the provision of large amounts of data. They also stress that data opening allows passengers and other road users use to gain a better travel experience and that this approach can be assessed as a financial/ economic contribution to customers and organizations. The usefulness and value of the open data is proved by a case study describing how one of the world’s largest public transport operations, Transport for London, transformed the availability of real-time of data, including live arrivals, timetables, air quality, network performance and accessibility, for its customers and personnel, using the open data approach, and what the results of this transformation were. Since these data are available for both commercial and non-commercial purposes, the data are already used by companies such as Waze, Twitter, Google, Apple, Citymapper, Bus Checker, Bus Times and Mapway, as well as academics and professional developers, to create over 600 customer-related products and services, reaching millions of active users, that enable passengers and other road users use to gain a better travel experience. The authors are sure that insights from the data can spur new thinking in Transport for London, boost network demand and improve customer satisfaction. They also highlight that interest in real-time open data has produced a quality feedback loop that has encouraged all data contributors within Transport for London to improve their data in terms of both granularity and quality. This success of a third-party app ecosystem has pushed Transport for London to make a strategic decision to work more closely with developers.

However, there are researchers who are not so positive and are of the opinion that, in some cases, more precisely in the case of COVID-19 OGD, it is not clear whether any data opened provides anything useful beyond adding to the reputations of governments for becoming more open and transparent [45]. In their analysis a case study of Canada is proposed to assess the value of open SARS-CoV-2 positive case numbers. Using a combination of real data and simulations, they found that daily publicly available test results may show significant errors in relation to individual risks (measured as a proportional part of positive, population-related and active cases) and that short-term differences are unlikely to provide useful information on reliable decision-making by individual citizens. They also urge governments opening data to highlight shortcomings of the data being opened to ensure that the public is properly informed about the uncertainties associated with SARS-CoV-2 information.

In general, the majority of studies found which actively utilize or promote open data can be classified in at least two general categories, where open data are used as (1) an input for new services, such as medicine or healthcare, transport, environment, smart city or smart home solutions, or (2) as a tool to improve the algorithms already developed, optimize solutions in use, or introduce new ones where the open data can be used as training data without the need for resources (both, time, money and human) to be spent on data collection. However, the way in which open (government) data are reused is very different, pointing to both (1) their potential by themselves, where data opening can be considered to be the key to various benefits, both commercial and non-commercial, and (2) their potential in regards to Society 5.0; and the more data become available, the more new application areas will be explored. This also points to the lack of studies on the connection (if any) of open (government) data, Society 5.0 and Industry 4.0 concepts, i.e., only a few studies are addressing this. In addition, there are few studies on the evaluation of the OGD portals and their data regarding today’s trends, which have now changed significantly, where open data should not only be available and accessible, but other “must have” aspects that are essential to meet users’ needs should also be identified and met. This paper attempts to establish such a study and encourages further studies in this direction.

## 4. Materials and Methods

This section deals with the method used to assess 60 countries and their national open data portals for their correspondence with Society 5.0 trends, as well as the results obtained when solving RQ3. The number of countries under consideration is explained in [19], as this article is an extended version. It includes 32 European countries covered by the European Maturity report (ODMR), with another 19 countries added from a previous study as countries with the earliest cases of COVID-19 [19].

In total, 60 countries were addressed during the study. For nine countries—Liechtenstein, China, Macau, Cambodia, Egypt, Lebanon, Afghanistan, Iraq, Kuwait—national open data portals have not been found. However, this does not mean that these countries do not support open data initiatives, since several country-related data sets can be found on the web (i.e., at least for Cambodia, Egypt, Lebanon, Kuwait and Afghanistan), but since OGD portals are the focus of this study, these countries are not addressed. Thus, 51 countries owning their own national open data portals were further addressed.

Each portal has been studied starting with a search for a data set of interest, i.e., “real-time”, “sensor” and “COVID-19”, followed by a list of additional questions. These questions were formulated on the basis of combination of (1) crucial open (government) data-related aspects, including open data principles, success factors, recent studies on the topic, PSI Directive [29] etc., (2) trends and features of Society 5.0 and Industry 4.0, and (3) elements of the Technology Acceptance Model (TAM) and the Unified Theory of Acceptance and Use Model (UTAUT). The later UTAUT model served as a source of inspiration for [2], the authors of which linked the concepts of OGD, Society 5.0 and Industry 4.0. Therefore, a brief overview of these models is unavoidable. The following subsections therefore refer to TAM and UTAUT in relation to this study, and are followed by a brief overview of the PSI Directive main points, which affect the list of aspects to be examined.

### 4.1. TAM and UTAUT Models

The desired output of this and similar studies was, is and will be the acceptance of the OGD portal together with data they provide as a technology. Thus, the Technology Acceptance Model (TAM) and its successor The Unified Theory of Acceptance and Use Model (UTAUT) should be considered to define some OGD portals and their data “must have” characteristics.

TAM consists of “perceived ease of use”, “perceived usefulness”, and “attitude towards use” resulting in “intention to use”, which should therefore result in “actual usage” [46], where (1) *perceived ease of use*, i.e., the amount of effort needed to use open data, can be linked to machine-readability and API presence in the case of OGD portals and their data; (2) *perceived usefulness* can be linked to a summary of how the data set was usable for other users, including statistics on the number of downloads, rating and reuses/showcases, comments (if any), and frequency of updates determining whether data may be used for a trusted output, and machine-readability together with API, allowing their use to assess what benefits could be gained from the use of open data; (3) *attitude towards use*, linked to the opportunity to create feedback, leave a comment, suggestion and/or complaint, along with its visibility to others and the ability to see whether this was taken into account, i.e., whether users’ opinion is considered as important. These points together are potentially able to affect intention to use and, as a result, to achieve the main goal—*actual usage* of the OGD portal and its open data sets. Thus, within the scope of this study, the final stages of the TAM model, i.e., “*intention to use*” and “*actual usage*”, serve not only as an objective to be achieved by governments within their OGD portals, but also as an aspect to be verified, i.e., whether the external variables have a direct impact on showcases or use-cases developed (considered as the evidence of “*actual usage*”), when these data are provided on the portal.

UTAUT is described as “performance expectancy”, “effort expectancy”, “social influence”, and “facilitating conditions”, resulting in “behavioral intention” leading to “use behavior” [47,48]. *Performance Expectancy* refers to individuals’ willingness/readiness to use the OGD portal and its data, if they believe that it could help them to generate higher incomes or to obtain some extrinsic benefits, which in turn would increase their expectancy to perform better professionally [48]. This comes with an insight into how the open data set was or was not useful to other users and is closely associated with (1) use-cases/showcases (if any), their number and their nature, (2) statistics on open data sets, i.e., number of downloads, number of reuses, rating, number of views etc., (3) in some cases, the feedback in terms of the comments is also beneficial, but only with a premise that the portal allows it to be seen by other users, i.e., not only by the author of the particular entry. *Effort Expectancy* refers to the ease or difficulty of using open data technologies, finding data, and the skills needed to retrieve and use the required data sets. If individuals perceive data to be easily accessible and they do not have to invest much in their use, their tendency to use these data increases [48]. Here, in addition to the preliminary results, i.e., whether the relevant data has been found, such aspects as their machine-readability and API matter, which simplify the use of data. Comments and other types of feedback may be linked indirectly because they can give the user insight into how the user is or is not supported. *Social Influence* implies the extent to which individuals perceive others to have a significant impact on their use of open (government) data [48]. It is also influenced indirectly by use-cases and feedback provided by other users, as well as statistics on the data usefulness able to affect users’ decision. *Facilitating Conditions* refer to the dependency of individual’s use and acceptance of OGD on the availability of the necessary infrastructure, such as internet access or appropriate data infrastructures [48]. Again, for OGD portal-related aspects, machine readability and API, which makes it easier to integrate open data with the data that the user is working with, and possible assistance/feedback, are important. This together forms *Behavioral Intention*, which can result in *Use Behavior*, which is considered to be the desired output.

### 4.2. PSI Directive and Open Data Directive

Another source for establishing criteria was the PSI Directive. In its recent report [29], the European Commission has underlined the importance of real-time data, enabling companies, especially startups, to develop innovative products and services such as mobility apps. However, in order to use these data effectively, [29] stresses the importance of API. Although this relates mainly to real-time data, this study will check the availability of API for all types of data since this is in line with Industry 4.0 and the future of open (government) data. Today, API is one of the most crucial factors for turning data into economic value because bringing open data closer to entrepreneurs and citizens influences open (government) data’s potential re-use for the creation of new services and products [3,38,44,49,50]. As an example, Aguilera et al. [49] stress that the API allowing real-time data contributes to the development of smart cities and urban applications, while, according to [44], the introduction of the API for Transport of London-related open data even caused a temporary halt, due to high demand by apps using this service. Aguilera et al. [49] stress that API allowing real-time data contributes to the development of smart cities and urban applications. To sum up, it is recognized as one of the best practices for data platforms.

### 4.3. Protocol

Now, let us sum up and present the methodology of this investigation. The method used belongs to typical/daily tasks of open data portals, sometimes called a “usability test”—keywords related to a research question are used to filter data sets, i.e., “*real-time*”, “*real time*” and “*real time*”, “*sensor*”, ”*COVID*”, “*COVID-19*”, “*corona*”, “*coronavirus*”, “*virus*”. In most cases, the keywords “*real-time*”, “*sensor*” and “*COVID*” were sufficient to select all data sets associated with the issue, but depending on the open data portal and its language, some additional keywords and translations in the respective languages were performed.

While some portals were user-friendly in filtering search results by the publishing date, other portals (a) were not able to filter data by publishing date (only “recent updates” or “most popular”), or (b) the search filter was not available at all. Another aspect is that many portals do not provide data on the date of data publishing, the frequency of updates, and the date of the most recent update, or do not force the data publisher to provide this, i.e., these data are not mandatory. The examination of the respective aspects for less user-friendly portals was adapted to particular cases based on the portal or data set specifics. Otherwise, the nature of the study mainly requires a repeat of the same simple actions on all portals being addressed, by checking:

1. *Are the open data related to the topic under question ({sensor; real-time; COVID-19}) published, i.e., available?*
2. *Are these data available in a machine-readable format*
3. *Are these data current, i.e., regularly updated?*

The criteria regarding currency depends on the nature of data, i.e., COVID-19 data on the number of cases per day is expected to be updated daily, which would not be sufficient for the real-time data the title supposes, etc. This means that each data set should be inspected and an “expected” update frequency should be determined, i.e., update frequency via which the data will be considered to be up-to-date, which is then compared with the actual. These data may sometimes be retrieved from the frequency of updates defined by the data publishers but do not always make sense. In addition, in some cases, even if the particular keyword was met but had a note that this data is for a certain period which has ended, the most recent updates contradicting the defined time frame have not been assessed;

4. *Is API ensured for these data (most importantly for real-time and sensor data)?*
5. *Have they been published in a timely manner?*

This was verified mainly for COVID-19 related data, since timely data has higher potential to be valuable for early deployment of different solutions bringing benefits to society. As an example, according to [24], the number of cases in Mainland China could have decreased by 66%, if the data collected had been opened just one week earlier. Timeliness is assessed by comparing the dates of the first case identified in a given country and the first release of open data on this topic.

In addition, some general aspects were examined in order to gain general information on the portal concerned, closely linked to a “human-oriented approach”:

6. *What is the total number of available data sets?*
7. *Does the open government data portal provides use-cases/showcases?*
8. *Does the open government portal provide an opportunity to gain insight into the popularity of the data, i.e., does the portal provide statistics of this nature, such as the number of views, downloads, reuses, rating, etc.?*
9. *Is there an opportunity to provide feedback, comment, suggestion or complaint?*
10. *Is the artifact, i.e., feedback, comment, suggestion or complaint, visible to other users?*

In the previous study [19], the case of Spain with regard to the COVID-19 open data was referred to as one of the best practices thanks to customer support, which led to active discussions between users and portal staff and the meeting of their requests within a short time. As part of the service quality, this is a major benefit/asset and even a driver for citizens’ trust in the OGD portals data [51]. Therefore, this study includes the relevant aspect as a question to be asked to all the portals analyzed, identifying how often (if at all) and how user interaction and support are promoted.

This set of questions (6–10) was applied to both the portal as a whole and to each particular keyword under consideration, thus allowing an insight into the popularity and trends of the portal in relation to the general state of the art.

One of the questions deserves more attention, i.e., *whether these data are really valuable for users?* This question can be answered by understanding whether the open data are being reused and is closely linked to the acceptance of the open (government) data, the OGD portal and UTAUT and TAP. Most portals do not provide the possibility of obtaining these data, but there are some portals that provide information about data usage in the way of number, and sometimes even the nature of the reuse, number of views and downloads. Therefore, the following actions are required: (1) determine whether the OGD portal provides a user with use-case/showcases, (2) determining what is the total number and nature of these use-cases, (3) determine the breakdown of these use-cases across the topics under consideration. In addition, the data set specific statistics (if any), i.e., the number of data set downloads, the number of views, the rating of the data set and/or reuse associated with a particular data set, will be examined. Although the previous study looked at these questions separately in a very brief manner, portals deal with them very differently and this would require considerable resources to obtain these data and process them uniformly; this time this analysis was conducted and the respective results will be presented.

The presence of the feature under consideration is assessed with a 3-point scale (−{1..1}), where “−1” is not fulfilled, “0” is not always fulfilled, “1” is fulfilled. Although in most cases Boolean with 1/0 (yes/no) is used for such tasks (for instance, [22] and some international rankings), this study involves another point, thereby improving flexibility in cases where doubts may arise, i.e., the majority of data sets meet the criteria, while there are some data sets, which do not. This is also due to the fact that OGD portals collect data from different publishers, so the portal cannot obtain a very critical rating if only one of many publishers ignores open data principles or does not follow other guidelines. However, in cases when binary scale is more appropriate, i.e., assessing whether the portal provides use-cases, any changes compared to other “typical” studies have not been involved.

All the questions addressed above were asked of each selected portal. As a result, a set of individual protocols in the form of Table 4 was collected.

Thus, the study concerns both data, impact, use and platform, where the last three aspects are considered as relatively rare due to the complexity of evaluation, being also very time-consuming due to the immaturity of open (government) data practices [22].

## 5. Results

As a result of the analysis of these portals and the resulting protocols, a joint protocol was obtained for all the countries examined (51 country × 3 topics), partly presented in the next subsections. The whole protocol is available online in Open Access [53], thereby supporting “open science” principles.

First, the general characteristics will be briefly addressed, which are briefly summarized in Table 5.

OGD portals might be very diverse, from the nature of data they provide their users with to the features supporting users’ interaction with the portal. This also applies to the *total number of data sets* provided, where the number of data sets on the portal may vary from 47 data sets for Greece to 291,274 for the United States (USA). This also applies to *reuses or showcases*, where the total number may vary from 3 for Bulgaria to 2658 for South Korea in addition to 63 visualizations and 95 “crowd-mapping”. This also points to the diversity of the nature of reuse, which can differ from simple static visualizations, which can also be dynamic, to complex applications and services. This continues with regards to *mechanisms indicating the popularity* or value of a particular data set, where it can be expressed as a rating of the number of views, the number of downloads, the number of reuses of a particular data set. In this respect, the most popular choice for developers of the OGD portal is the provision of a number of views per data set (14 of 51), followed by ratings (12), the number of downloads (8), and then the least popular, but perhaps the most expressive, i.e., reuse (4), which is available on the portal of France, Ireland, Austria and Portugal. The majority of portals ensure a combination of multiple opportunities. Unfortunately, 28 OGD portals do not provide any features of this nature.

Only 25 out of 51 OGD portals (49.02%) provide an *opportunity to provide feedback, comment, suggestion or complaint*, and two only allow this after logging, which is not in line with best practice, as this should be allowed by all stakeholders without additional activities such as registration. In terms of the visibility of these artifacts, the results are even worse, because only 17 OGD portals (33.33%) ensure their visibility to other visitors, and three require logging into an account. This means that most portals do not support active interaction and feedback with users, which should have a positive impact on both the OGD portal and its content, the intention of users to use the portal and the data it provides, which could result in further reuse.

The following sections address each particular data category concerned.

### 5.1. Real-Time Open Data

**Figure 2:**
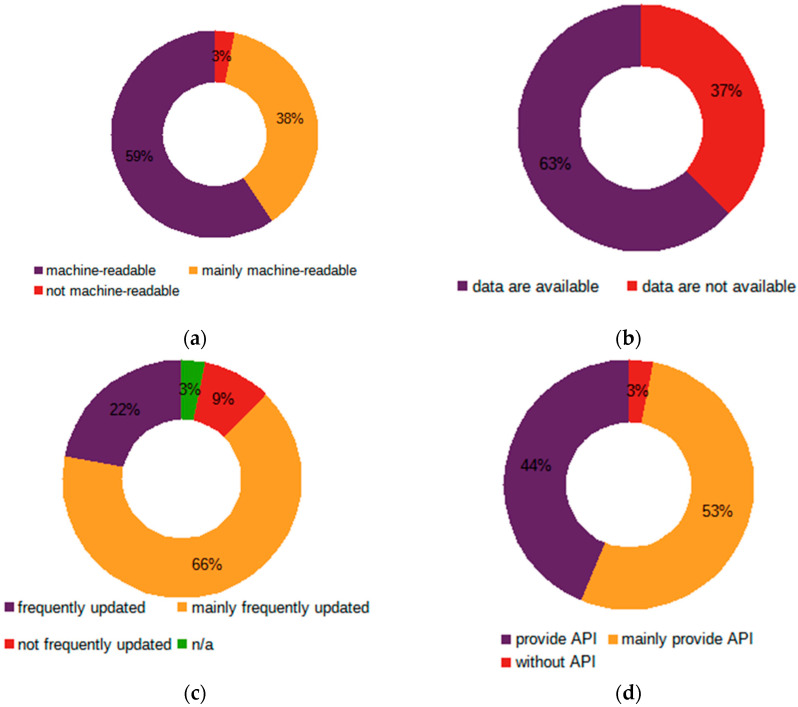
(**a**) Real-time data availability on 51 OGD portals; (**b**) Real-time data machine-readability; (**c**) Real-time data currency, i.e., frequency of updates; (**d**) API presence.

Real-time data amounts are significantly higher compared to static data, so a mechanism is needed that deals with them and allows their retrieval into an external service such as app. This is usually done by enabling data to be obtained through the API. Therefore, this section focuses on this specific aspect, in addition to other “must have” aspects discussed earlier (Figure 2).

For general statistics, 32 out of 51 OGD portals provide real-time data with a total of 19,437 data sets, with at least one data set for India, the Netherlands, and Latvia and 9449 data sets for USA. Although this number may sound impressive, if the total number of these data sets is compared to the total number of all open data sets available, the ratio is less than 2%, more precisely just 1.87%, which does not correspond to [29] expectations. Furthermore, the number of data sets for which the mandatory prerequisite of API is ensured is even less, not to mention the fulfillment of all the requirements.

For data currency, i.e., whether they are up-to-date, although the name of this category supposes that all data must be updated continuously, the results do not correspond to this assumption. This is mainly because some data sets falling under this category are no longer updated. Some datasets, despite promising “real-time” data, are neither real-time data nor nearly real-time data.

To sum up, only 7 OGD portals, namely France, the Netherlands, Luxembourg, Portugal, India, USA and South Korea, meet this requirement. Six more countries face some challenges in this respect, i.e., some data sets cannot be considered as real-time data, while the majority are recognized as such; 21 face more significant challenges and a further three fail.

As regards their machine-readability, more than half of the OGD portals (19, or 59.38%), provide data in machine-readable format with 12 OGD portals facing challenges for individual data sets, where for five of them the number of machine-readable data sets significantly dominate, with one fail (Japan).

A surprising but similar trend occurs in the case of API, which, according to good practice and the PSI Directive, is a prerequisite for real-time data, but not all OGD portals provide an opportunity to retrieve the data through an API or via a similar approach, thereby reducing the overall value of these data, i.e., preventing them from being managed quickly and effectively.

However, despite many countries and portals facing challenges in providing machine-readable, easily retrievable real-time data, these data are extremely popular for further reuse. At this point, 19 portals provide 302 reuses/showcases and numerous visualizations, with the highest number of reuses provided by South Korea, followed by Spain, Sweden and France. They may vary in terms of their nature, but the most popular reuses, just like real-time data sets, are related to traffic, air quality, meteorology/weather, climate, and noise levels, with a strong focus on the Smart City.

Table 6 provide the TOP-10 OGD portals obtained by combining data machine-readability, presence of API for respective data sets and their currency, i.e., frequency of updates (for the whole list see [53] dataset).

It can be concluded that, although there are portals working on their content and functionality and trying to move towards being “smarter” and meeting Society 5.0. expectations, at this point the majority of the OGD portals are far away from gaining real value from open data, though many portals accept them as data of high value.

### 5.2. Sensor-Generated Open Data

As regards sensor-generated open data, the analysis has revealed that out of 51 OGD portals analyzed only 29 OGD portals (56.9%) provide sensor open data, with a total number of 23722 data sets, where the number of open data sets per portal vary from 1 for Latvia to 18,530 for USA.

As for currency, only 5 OGD portals do not face challenges in this respect, providing data that are updated in a timely manner or at least as often as the data provider has promised in the “frequency of updates” or relevant field. Another 17 OGD portals experience some problems, i.e., not all sensor data are updated frequently, among which even such leading countries in terms of open (government) data as France and Ireland are listed, and seven portals face significant challenges.

**Figure 3:**
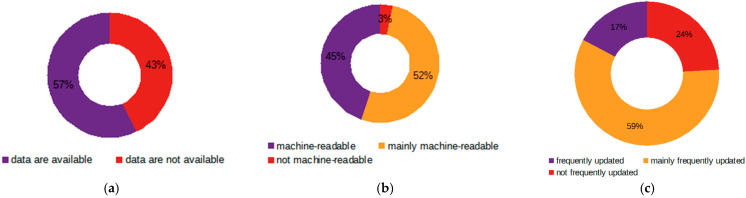
(**a**) Sensor data availability on 51 OGD portals; (**b**) Sensor data machine-readability; (**c**) Sensor data currency, i.e., frequency of updates.

Regarding machine-readability, which could be considered as a prerequisite for the reuse of sensor data, the results are much better compared to currency; 13 OGD portals provide their users with machine-readable data, and 15 OGD portals, mostly machine-readable with only 1 OGD portal (Japan), face challenges in this respect. Figure 3 provides an overview of the current state of OGD portals in terms of sensor data.

Table 7: TOP-10 OGD portals.

To sum up, even those countries that provide their users with sensor-generated data are often unable to provide these in accordance with open data principles (see Table 7 and the wcole protocol in [53]), facing challenges in terms of their machine-readability and even greater problems with their currency, i.e., the frequency of updates. Surprisingly, only a few portals (6 out of 29) have use-cases where data of this type are reused.

### 5.3. COVID-19 Open Data

A previous study revealed that only 32 OGD portals analyzed provided their users with COVID-19-related open data, but at this point this number has increased to 40, as another eight countries, i.e., Slovenia, Romania, Greece, Denmark, Bulgaria, Portugal, Japan and Philippines, have also published COVID-19 related open data. Thus, some countries which have previously demonstrated unexpected results (Finland, Slovenia, Romania and Greece) despite being considered sufficiently competitive in terms of their OGD portals’ data [19] but not yet publishing COVID-19-related open data, have now published these, with the exception of Finland, which has not yet released them on the OGD portal. Thus, the ratio of countries providing COVID-19-related open data to their users has increased to 78.4%, and allows us to argue that the vast majority of OGD portals provide this type of data. However, the analysis of the respective data sets has shown that in some cases the data sets are not focused on the numbers of cases, vaccination and its progress, etc., but on modifying other non-pandemic related data that have been affected by the causes of the pandemic.

These data sets are also the most popular on the majority of the OGD portals, since they provide probably the most urgent and crucial current data. This applies to the individual OGD portals of the countries under consideration but also to the EU Open Data Portal (data.europa.eu), which has collected a total of 849 data sets and eight use-cases/showcases by mid-August 2020, which had increased to 1932 data sets and 28 use-cases by June 2021. This trend is also observed in this study, i.e., the previous examination of OGD portals found a total of 861 data sets focused on or affected by COVID-19, while in June 2021 their number increased by 59.52% to 2127 data sets with at least one data set for Romania and the highest result of 505 for USA.

In many countries, OGD portal holders and researchers have noticed an increase in the number of frequency of visits to the OGD portal with the opening up of these data. This can be explained through the fact that opening up data and models that are the basis for decision-making on the COVID-19 pandemic (or any other issue) enable people to understand the decisions, scientists to scrutinize and improve them, and neighboring countries to support each other [54]. Of course, data reliability is a matter of no less importance, but this paper addresses timeliness and data availability, leaving this question for other studies. Timeliness is defined as “made available as quickly as necessary to preserve the value of the data” [55]. However, reality shows that this is a challenge for data publishers and portal holders, although this affects user’s intention to use open data and the all open data portal [56,57,58,59,60,61,62,63,64]—users become less likely to create open data-based services if a lack of this principle is observed [65].

The question—have the COVID-19-related data been published in a timely manner?—was broken down into (a) data mentioning COVID-19, (b) country-specific exact data on COVID-19*,* as it was observed that many data sets containing “Covid-19” as a tag or keyword relate to other non-medicine-related topics, such as data sets on restaurants delivering meals, traffic changes, etc., which is in line with data.europa.eu, according to which the data available on COVID-19 ranges from epidemiological, healthcare facility and medical research to data on governmental measures and the socioeconomic and environmental impacts of the pandemic. Thus, it was not enough to find data with a matching keyword and then sort it by the date of publishing, and additional investigation was necessary.

**Figure 4:**
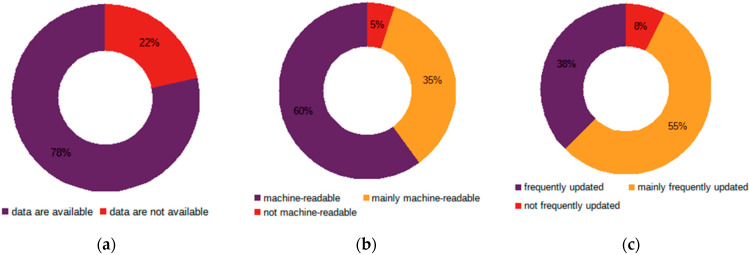
(**a**) COVID-19 data availability on the OGD portals; (**b**) COVID-19 data machine-readability; (**c**) COVID-19 data currency, i.e., frequency of updates.

This examination resulted in the discovery that only a few countries published appropriate data in a timely manner. The nature of the data opened is mainly related to the number of cases identified, the data on these cases, the number of tests (in some cases these were added later, when the activity took place) and the results of these tests. The nature of these data has now been complemented by data sets on vaccination. Austria, France, Switzerland and the USA are countries that have published relevant data in the first two weeks (7.7% of 52 portals and 6.7% of 60 countries), while five countries, Estonia, Colombia, Latvia, Cyprus and Ireland, opened them in the first month, 14 countries have done this in more than a month (26.9% or 23.3% of 60 countries), and for other countries these data cannot be identified (see Figure 4) since these portals have not provided appropriate data (in the scope of specific data sets or because they do not provide these data at all). This is the case even for such competitive countries as Spain, Finland, Netherlands, and Italy. As for European countries, the best results were demonstrated by Austria, France, Switzerland, Estonia, Latvia, and Cyprus.

For the most competitive countries in terms of data release, these results mainly correspond to [66], where all these countries gained from 2.63 to 3 out of 3 points (corresponding to “fulfilled”) and only the USA gained 2.33 and Ireland 2.55 for the relevant *“release date and up to date”* aspect. At the same time, the countries that published COVID-19-related data in a month after the first case were mainly assessed with 1.84 to 2.22 points, with the exception of Luxembourg, Germany, Canada and Taiwan, which gained rather high results in [66], but demonstrating a delayed response in times of an emergency/pandemic.

Another interesting point is that 14 countries have published some data sets, partly related to COVID-19, or informative materials before more specific data sets aimed to provide data on the current state in the specific country that can be further reused, producing valuable results. For four countries, Spain, France, Cyprus and Switzerland, this is a positive example of managing open data, because they have adapted/updated already existing data sets with COVID-19 data, since the pandemic affected a number of aspects of everyday life, and in many cases data on such topics as public places, traffic and other were also affected. However, although this is a positive point in most countries, since not only the statistical data sets but other valuable data sources were also published, in three cases—Poland, Denmark, New Zealand—data of that nature only are available.

The positive point is that nearly all published data are in a machine-readable format and can be easily accessed and processed without additional actions, so in most cases there are no barriers to reusing data, as 24 countries provide data in a machine-readable format. Other countries—Poland, Germany, Greece, Croatia, Estonia, United Kingdom (UK), USA, Australia—sometimes experience difficulties with machine-readability of open data, where Belgium, Switzerland, Portugal, Lithuania, South Korea and Iran have demonstrated slightly better results (the ranking system has not allowed to demonstrate it) and another two countries, Japan and Denmark, face bigger challenges, which means that sometimes *.html* to other resources and *.pdf* are provided without machine-readable files, making the re-use of data extremely challenging. However, this can be explained by the fact that these portals have published not only data but also related external links and laws, as well as news, to inform their citizen as much as possible on the matter. Compared to [29], some portals, USA, Estonia, Colombia, Latvia, Lithuania, Luxembourg, Sweden, Germany, Croatia, Australia, Taiwan and New Zealand, demonstrated even better results, while according to [29] this aspect is a challenge for these countries.

15 countries provide users with up to date data by which is meant that they are regularly updated, while some countries face some challenges (Iran, India, South Korea, United Kingdom) and for three countries—New Zealand, Japan and Denmark—this is a big challenge. A positive point is that the number of countries facing significant challenges in this respect has decreased from six countries to three, i.e., Taiwan, Lithuania, Poland and Austria have improved their results. Overall, given that this aspect is usually the most complicated for almost all open data, the results obtained are relatively good, but still far from very positive, with only 37.5% providing up-to-date data, and 40% OGD portals working hard on it.

As an example, in the case of Latvia, data on new cases, tests, the number of sick (their age, city, etc.) and complexity of cases appear more quickly than on news portals—it seems that news portals retrieve data from the OGD portal, thus the OGD portal becomes the most up-to-date tool to track the situation in the country. According to the number of views and the associated filter “by popularity”, both COVID-19-related data sets are the most popular data sets on the portal, so the society has noticed and uses them. Unfortunately, there is a lack of information about the nature of the reuse of these data, which is also the case for other countries.

A summary of the most competitive countries in terms of this study is provided in Table 8 (see the whole protocol in [53]). In the case of COVID-19 data, the number of competitive countries is much higher compared to the above categories, i.e., 19.

As regards the popularity of the COVID-19 open data provided by the portals, a total of 312 use-cases are provided by 19 portals (59.38%), with the highest result for France, which provides 205 COVID-19 open data-based use-cases, followed by India, Colombia, Netherlands, Sweden and Romania. The number of countries reusing COVID-19 open data has increased significantly, since the previous study [19] revealed only a few, more precisely, Poland, Austria, Cyprus, Switzerland, Spain, Taiwan and France, and the number of reuses provided by the French OGD portal has increased twofold. This, however, can be easily explained by an excellent opportunity provided by the portal to users—a tool that allows stakeholders to upload their use-case, thereby facilitating user participation. Taking a step back to the protocol provided in Table 8, it can be seen that France was among the countries that supplied COVID-19-related data to the audience as soon as possible and the first occurrence of the data was five days after the first case identified in the country.

As for other countries, data in Austria and Switzerland were also made available in a timely manner of less than a week. However, the open data of Taiwan and Luxembourg became available with a delay, but, nevertheless, in terms of the pandemic and its harmful nature, the audience was attracted to this data. In addition, the fact that these data became available not immediately after the first case identified does not mean that these data are not up to date.

It should also be emphasized that the lack of information on the reuse of open data does not mean that these data are not re-used, as is sometimes concluded. One of the examples here is the Latvian COVID-19-related open data, which, despite the lack of information on reuse in terms of specific use-cases, are used at both national and international levels, including the contact tracking application *“Apturi* *COVID” (“Stop* *COVID”),* which obtains statistical data from Latvia’s open data portal. 

This demonstrates public interest in open data, which means that open (government) data is not only a modern trend but also a powerful tool valuable to both government and society that is also in line with [67]. In addition, this extended study supports previous findings [19], according to which, in many cases, the quality of the data associated with the pandemic, i.e., COVID-19, is higher. In this study this is related not only to machine-readability and frequency of updates for certain countries but also to the availability of data of this type compared to real-time and sensor data and the number of use-cases provided by these portals.

## 6. Discussion and Limitations

### 6.1. Results and Discussion

To sum up, out of 51 national open data portals, 40 OGD portals provide open data related to COVID-19, 32 portals provide real-time data and 29 provide sensor data. This means that while many countries are trying to follow the latest trends and provide data that could be important for their users in transforming into innovative solutions and services that create added value for both the economy and the society, including moving towards the Smart City and the Smart Society, some countries have not yet opened these data. Although in general, “smarter” data and higher quality data are often typical of highly developed countries, which follow the trends of Smart Cities at both economic, political and social levels, for many countries this relationship is less obvious, i.e., developed countries can demonstrate weak results in terms of data provision and their usability, while less developed countries can be characterized by relatively competitive results. Even more, among the countries that have already opened these data, the majority of portals have gaps in the usability of these data in terms of their machine-readability, the unavailability of API, and the timeliness and frequency of updates.

Many countries also do not support their own users by making support mechanisms available: only for less than 46% of portals, with an even smaller number of portals promoting this cooperation transparently, i.e., to be seen by others, and therefore their users may be less likely to use the data and the whole portal. However, most OGD portals provide data in a machine-readable format, while some countries sometimes face challenges in this respect. For some countries this is due to the fact that these portals publish not only data but also related external links and laws, as well as news to inform their citizens of as much as possible of topics of potential interest.

Some OGD portals were found to have a delayed response during the pandemic compared to a normal situation without emergency. However, in some aspects, such as machine-readability, most OGD portals demonstrated significantly better results, i.e., COVID-19-related data were provided in an easy to process format, while often this aspect is a challenge for some countries.

It is surprising that real-time data are not always updated continuously and cannot always be used effectively, but their popularity is increasing and their value to users is noticeable in terms of the number of reuses published on the OGD portals, using data dealing with such crucial topics as environment and daily routines such as traffic. However, despite relatively low results, these data are still of high interest for users as demonstrate by the number and nature of reuses available on the OGD portals, and a literature review that looked at some of the other reuses of real-time data, demonstrating some kind of value and benefits they can provide to both citizens, economy and science.

### 6.2. Limitations and Future Directions

The analysis of open data by topic, i.e., real-time and sensor data, and OGD portal features examined in this paper is consistent with the trends in Industry 4.0 and Society 5.0 [2,68,69,70]. However, this study has some limitations, at both the study level and in completeness of scope in terms of Society 5.0 and Industry 4.0.

First, given that the analysis was performed by means of a typical task, i.e., searching for a data set/use-case using keywords, it can be argued that the results may not be very accurate. This may be related to several facts: (1) the presence of a matching keyword does not guarantee that the data set is indeed linked to that keyword, as was observed for COVID-19 data, i.e., the presence of a keyword can be associated with a description of an unrelated data set whose content was affected by the effects of the pandemic, (2) some data sets may not be found if the data publisher has not provided a sufficiently detailed data set title, description, tags and metadata. The second issue has been discussed in [66], where it has been revealed that it is usual for OGD portals, in the case of categories assigned to a data set, not to assign or to assign incorrectly or without adequate/sufficient description of a data set, such as a recopied title. This could affect the results, but the objective was to examine typical actions done by users searching for data, so the results can be considered to be accurate with regard to this prerequisite, i.e., data sets that are not found within the scope of the analysis correspond to the result that the user will receive interacting with the portal.

Secondly, although preference was given to a three-point scale to provide more flexibility when assessing the presence of specific aspects rather than a very limited binary scale (yes/no), it is still not enough to allow the ranking of different countries as regards the level and quality of meeting specific criteria. Although this study does not aim to rank the countries, thus gaining an overall insight into whether and how countries make steps toward the Society 5.0. paradigm, in the case of similar research projects with the aim of ranking countries, a larger scale would be beneficial, especially when frequency of updates are evaluated, to allow for better breakdown of results. However, although this would allow more accurate results with further possibility of cross-country comparison, this would require controlling for the reliability of the results. This means that additional mechanisms should be considered, or the involvement of a team of researchers conducting such an analysis, thereby avoiding bias. However, the scale used in this study avoided this, since the presence and absence of artifacts were mainly assessed by “−1” or “1”, but in the case of some deviations “0” points have been assigned. Weights can also be assigned to certain aspects, such as machine-readability, API and timeliness.

In addition, the assessment of open data may include more aspects compared to those covered by this paper, e.g., an open license, the possibility of bulk download and metadata, both in terms of their presence, completeness and relevance to actual data. Some of the aspects covered could be extended, e.g., by analyzing in depth the machine-readable formats used for open data sets (such as xls, xml, csv, shp, etc.) and their correlation (if any) with the re-use of these data. This study did not intend to cover the open license issue, given, rather, that it related to data compliance with open data principles, focusing on principles that ensure the re-use of data. However, the observations made during this study show that this aspect should be further inspected. Although the latest studies highlight that the open license is no longer an issue for open (government) data and there is a common assumption that open license is ensured by default, different licenses assigned to the open data sets have been observed, which sometimes limit the re-use of data. However, the metadata-related point also corresponds to the data.europa.eu (European Data Portal), according to which none of the 95,145 article-related data sets (15,886 sensor-generated, 73,348 real-time and 5911 COVID-19 data sets) has been recognized as a data set whose metadata would be of excellent quality, with only 1311 data sets assessed as “good+” (numbers obtained at the end of May 2021).

Thirdly, in the case of open data, the quality and reliability of the data is not of least importance. There is a number of studies on this issue, where one of the new studies related to the topic [45] points to inaccuracies in the OGD pandemic data with low short-term decision-making value. The authors therefore encourage data publishers to highlight the uncertainties associated with these data, because while the OGD can increase government transparency and accountability, it is essential to ensure that the public is properly informed about the uncertainty associated with these data. Although an in-depth analysis on this has not been carried out, a brief observation allows us to state that this is rarely done.

Fourth, the management of sensor-generated data involves collection and analysis of huge amounts of data from a variety of sources [43], which usually necessitates specific solutions to be involved at the end of the OGD portal in order to ensure their effective management. This is also sometimes associated with the concept of Big Open Data. Thus, portals that do not have sensor-generated and real-time data could be explored in regards to their technical capabilities that could explain the lack of these data, i.e., if such mechanisms are not in place. In addition, an in-depth analysis not only of the data but also of their re-use, including both the main idea and the techniques applied, has the potential to determine how closely open (government) data and Industry 4.0 are related.

Fifth, the study focused exclusively on OGD portals, but many countries today have favored a new Smart City portal concept that could have a closer link to the concepts in question, both in terms of open data subtopics covered and in terms of Society 5.0 and Industry 4.0.

Last, but not least, this paper links the concepts of open (government) data to Society 5.0 and Industry 4.0, but only a limited set of the common concepts were addressed, though covering both connection “categories”, i.e., “Human-oriented action”, “Sustainable development” and “Physical-to-digital-to-physical loop”. At present, knowledge of the interconnection points is very limited, so in the future, when these are determined, a more in-depth analysis could be performed, with a stronger focus on TAM and UTAUT.

## 7. Conclusions

The main idea of using open (government) data is to create economic value by using the data in a variety of ways and by different users, which is considered to be the basis for the idea of creating a “society of imagination” or Society 5.0 [2,12]. Although the majority of researchers study the impact of open data on society’s performance and the factors that improve the use of open data, given that open data stem from the Society 5.0 concept, this study examines whether the current state of the OGD portals and their data complies with these concepts and contributes to them.

The study has posed and answered three main research questions. First, a close connection of the concepts Society 5.0, Industry and open (government) data has been established. The potential of open (government) data was then identified for three selected open data subtopics, namely real-time, sensor-generated and COVID-19 data. The answer to both questions is based mainly on the review of the literature. The third research question was answered by an analysis of 60 countries, by examining the 51 OGD portals on the presence of the relevant data and their suitability for further reuse, by analyzing their machine-readability, currency or frequency of updates, the ability to submit request/comment/complaint/suggestion and their visibility to other users, and the ability to assess the value of these data assessed by others, i.e., rating, reuse, comments, etc., which is usually considered to be a very time-consuming and complex task, and therefore rarely conducted [22]. The analysis leads to the conclusion that although many OGD portals and data publishers are working hard to make open data a useful tool moving towards Industry 4.0 and Society 5.0, many portals do not even respect the principles of open data, such as machine-readability. Moreover, according to the lists of most competitive countries by topic, there are no leaders who provide their users with excellent data and service, therefore there is room for improvements for all portals.

Although the results on data set-related aspects cannot be generalized because only three types of data (which may overlap) have been examined, nevertheless, this shows the trends in the OGD portals. The results of a very simple analysis show that even a crisis, i.e., a pandemic, cannot compel OGD portals to publish the data that complies with all open data principles. The term “real-time” does not guarantee that data are actually provided in real time and can easily be retrieved to an external solution using API. More than half of inspected OGD portals do not provide support mechanisms for their users, and even fewer portals promote this cooperation transparently, i.e., to be seen by others. However, there are countries whose portals are sufficiently competitive and they demonstrate very promising results in moving towards the Society 5.0 paradigm.

The paper shows that open data, particularly those published and updated in time, are provided in machine-readable format and support to their users, attract audience interest and are used to develop solutions that benefit the entire society (the case in France, Spain, Cyprus, the Netherlands, Taiwan, Austria, Switzerland, etc.). Thus, the publication of open data should be done not only because it is a modern trend, but also because it incentivizes scientists, researchers and enthusiasts to reuse the data by transforming it into knowledge and value, providing solutions, improving the world, and moving towards Society 5.0 or the super smart society.

The findings of the study could be valuable to both OGD portal holders and organizations developing open (government) data guidelines, gaining insight into the aspects to be taken into account to ensure that their OGD portals meet today’s needs, data publishers prepare the data to be published ensuring a greater likelihood of their re-use, following modern trends, and data users gain general insight into the state of the current data and their potential. Given that there are currently only a few studies on open (government) data in the context of Society 5.0 and Industry 4.0, the study could urge new research in this area, i.e., of interest to researchers. The topic has a number of future directions that could benefit society on the way to Society 5.0.

## Figures and Tables

**Table 1:** Digital libraries used for literature review.

Digital Library	URL
ACM Digital Library	https://dl.acm.org/
IEEE Xplore	https://ieeexplore.ieee.org/Xplore/home.jsp
Science Direct	https://www.sciencedirect.com/
Emerald Insight	https://www.emerald.com/insight/

**Table 2:** Queries by digital library for real-time related open (government) data studies.

Title 1	Title 2
ACM Digital Library	Title: ((“open data” OR “open government data” OR “OGD”)) AND Title: ((“real time” OR “realtime” OR “real-time”)) OR Abstract: ((“open data” OR “open government data” OR “OGD”)) AND Abstract: ((“real time” OR “realtime” OR “real-time”))
IEEE Xplore	(“Abstract”:”open data” OR “Abstract”:”OGD” OR “Abstract”:”open government data”) AND (“Abstract”:”real time” OR “Abstract”:”realtime” OR “Abstract”:”real-time”) OR (“Publication Title”:”open data” OR “Publication Title”:”OGD” OR “Publication Title”:”open government data”) AND (“Publication Title”:”index” OR “Publication Title”:”real time” OR “Publication Title”:”realtime” OR “Publication Title”:”real-time”)
Science Direct	(“open data” OR “open government data” OR “OGD”) AND (“real time” OR “realtime” OR “real-time”) in “Title, abstract or author-specified keywords”
Emerald Insight	title:”(open data OR open government data OR OGD) AND (real time OR realtime OR real-time)” OR (abstract:”(open data OR open government data OR OGD) AND (real time OR realtime OR real-time)”)

**Table 3:** Popularity of the topic in the digital library.

Digital Library	O(G)D	Sensor			Real-Time			COVID-19		
		Total	Primary	Ratio	Total	Primary	Ratio	Total	Primary	Ratio
ACM Digital Library	961	375	61	6.35%	745	181	0.19%	61	4	0.42%
IEEE Xplore	1308	343	102	7.8%	350	60	0.05%	15	8	0.61%
Science Direct	224	310	43	1.85%	618	86	0.04%	35	19	0.81%
Emerald Insight	154	24	1	0.65%	4	2	0.01%	2	0	0

**Table 4:** Protocol.

Aspect Examined/Question Asked	Value Type
**General**	
Total number of data sets	number
Whether the portal allows statistics on the popularity of data sets, i.e., number of views, downloads, reuses, rating etc.	Boolean + nature
Whether the opportunity to provide a feedback, comment, suggestion or complaint is provided?	Boolean + nature
Whether the feedback, comment, suggestion or complaint left for data set is visible for other users?	Boolean
Are showcases/use-cases available?	Boolean + number
**Real-time** **open government data**	
Are real-time generated open data available on the OGD portal? What is the total number of real-time open data sets?	Boolean + number * * if the first answer is = 1
Are real-time generated data sets updated frequently?	{−1, 0, 1}—1—yes/0—not always/−1—no
Are real-time generated data sets provided in a machine-readable format?	{−1, 0, 1}—1—yes/0—not always/−1—no
Is API available?	{−1, 0, 1}—1—yes/0—not always/−1—no
Are showcases/use-cases available? How many?	Boolean + total number
**Sensor generated open government data**	
Are sensor generated data available on the OGD portal?What is the total number of sensor generated open data sets?	Boolean + number * * if the first answer is = 1
Are sensor generated data sets updated frequently?	{−1, 0, 1}—1—yes/0—not always/−1—no
Are sensor generated data sets provided in a machine-readable format?	{−1, 0, 1}—1—yes/0—not always/−1—no
Is API available?	{−1, 0, 1}—1—yes/0—not always/−1—no
Are showcases/use-cases available? How many?	Boolean + total number
**COVID-19**	
When was 1st case of COVID-19 identified? Where the data on the 1st case is obtain from [52]	date
Are the open data on COVID-19 available on the OGD portal? What is the total number of COVID-19-related open data sets?	Boolean + number * * if the first answer is = 1
When was the term COVID-19 first mentioned in OGD portals data set?	date
When was the 1st COVID-19-related open data set released?	date
Were the data published in a timely manner? Comparison of the first case (FC) against open data availability (ODA)—date of the release of the first data set * * comparison of [52] and OGD portal	{−1,0,1}—1—“in less than 2 weeks”, 0—“in a month”,−1—“more than in a month”
Are COVID-19 related data sets provided in a machine-readable format?	{−1, 0, 1}—1—yes/0—not always/−1—no
Are COVID-19 related data sets updated frequently?	{−1, 0, 1}—1—yes/0—not always/−1—no
Is API available?	{−1, 0, 1}—1—yes/0—not always/−1—no
Are showcases/use-cases available? How many?	Boolean + total number

**Table 5:** General protocol.

Country	OGD Portal URL	Datasets Total #	Statistics—Number of Views, Downloads, Reuses, Rating etc. (1/0)	Opportunity to Provide a Feedback, Comment, Suggestion or Complaint (1/0)	Show-Cases (1/0)
France	https://www.data.gouv.fr/	37,003	1	1 (0/1) ^1^	1
Spain	https://datos.gob.es/en	46,973	1	1	1
Ireland	https://data.gov.ie/	10,440	1	1	1
Cyprus	https://www.data.gov.cy/	1103	1	1	1
Finland	https://www.avoindata.fi/	1862	1	1	1
Slovenia	https://podatki.gov.si/	4762	1	1	1
Austria	https://www.data.gv.at/	30,653	1	1	1
Romania	https://data.gov.ro/	2324	1	1	1
Luxembourg	https://data.public.lu/	1386	1	1	1
Netherlands	https://data.overheid.nl/	16,143	**0**	1	1
Latvia	https://data.gov.lv/	490	1	1	**0**
Poland	https://dane.gov.pl/en	1437	1	1	1
Italy	https://dati.gov.it/	45,326	**0**	**0**	**0**
Germany	https://www.govdata.de/	47,901	**0**	1	**0**
Greece	https://www.data.gov.gr/	47	**0**	**0**	**0**
Croatia	https://data.gov.hr/	1175	1	1	1
Belgium	https://data.gov.be/en	13,445	**0**	**0**	1
Estonia	https://www.rik.ee/en/open-data	789	**0**	1	1
Denmark	https://www.opendata.dk/	846	**0**	**0**	**0**
Norway	https://data.norge.no/	1177	**0**	**0**	1
Bulgaria	https://data.egov.bg/	10,170	**0**	**0**	1
UK	https://data.gov.uk/	30,632	**0**	**0**	**0**
Malta	https://open.data.gov.mt/	205	**0**	**0**	**0**
Switzerland	https://opendata.swiss/en/	5878	**0**	1	1
Portugal	https://dados.gov.pt/en/	4333	1	1	1
Sweden	https://www.dataportal.se/	7245	**0**	1	**0**
Lithuania	https://data.gov.lt/?lang=en	1135	1	1 (0/1) ^1^	1
Czech Republic	https://data.gov.cz/english/	136,070	**0**	**0**	1
Slovakia	https://data.gov.sk/en/	2360	1	**0**	1
Hungary	http://www.opendata.hu/	58	**0**	**0**	**0**
Iceland	https://opingogn.is/	112	**0**	**0**	**0**
Russia	https://data.gov.ru/	23,729	**0**	1 (0/1) ^1^	1 (0/1) ^1^
Taiwan	https://data.gov.tw/en/	49,621	1	1	1
Canada	https://open.canada.ca/en/open-data	85,987	1	1	**0**
Colombia	https://www.datos.gov.co/	55,24	1	1	1
New Zealand	https://www.data.govt.nz/	28,192	**0**	**0**	1
India	https://data.gov.in/	n/a, 10,397 catalogues	1		1
USA	https://www.data.gov/	291,274	**0**	1	1
Singapore	https://data.gov.sg/	1893	**0**	**0**	1
Australia	https://data.gov.au/	n/a	**0**	1	**0**
Japan	https://www.data.go.jp/	27,168	**0**	**0**	**0**
Thailand	https://data.go.th/	2966	**0**	**0**	1
South Korea	https://www.data.go.kr/en/index.do	57,032	1	**0**	1
Hong Kong	https://data.gov.hk/en/	n/a	**0**	**0**	1
Sri Lanka	http://www.data.gov.lk/	136	**0**	**0**	**0**
UAE	https://addata.gov.ae/	2286	1	**0**	1
Philippines	https://data.gov.ph/	318	**0**	**0**	**0**
Iran	https://iranopendata.org/	770	**0**	**0**	**0**
Israel	https://data.gov.il/	915	1	**0**	**0**
Bahrain	https://www.data.gov.bh/	n/a	**0**	**0**	1
Oman	https://data.gov.om/	n/a	**0**	**0**	1

^1^ only for system users, i.e., registered users.

**Table 6:** Real-time data: TOP-10 OGD portals.

Country	Real-Time Data 0/1	Updated Frequently	Machine-Readable Format	API	# Of Use-Cases
Luxembourg	1	1	1	1	15
India	1	1	1	1	2 visual + 3 app
South Korea	1	1	1	1	100
France	1	1	1	**0**	34
Netherlands	1	1	1	**0**	**0**
Estonia	1	**0**	1	1	**0**
Switzerland	1	**0**	1	1	2
Portugal	1	1	1	0	**0**
New Zealand	1	**0**	1	1	2
USA	1	1	**0**	1	**0**

**Table 7:** Sensor data: TOP-10 OGD portals.

Country	Sensor Data 0/1	Total Number of Sensor	Updated Frequently	Machine-Readable Format	API	# Of Use-Cases
Slovenia	1	2	1	1	1	**0**
Luxembourg	1	2	1	1	1	1
Colombia	1	15	1	1	1	4
Hong Kong	1	4	1	1	1	**0**
Netherlands	1	25	**0**	1	1	**0**
Latvia	1	1	**0**	1	1	**0**
Germany	1	20	**0**	1	1	**0**
Spain	1	188	**0**	1	**0**	7
Ireland	1	38	**0**	1	**0**	**0**
Finland	1	9	**0**	**0**	1	1

**Table 8:** COVID-19 data: TOP-10 OGD portals.

Country	1st Case of COVID-19	1st Mentioning in OGD	1st COVID Data Set	Total # of Data Sets	Machine-Readable Format	Updated Frequently	API	# Use-Cases
Romania	26.02.	15.02.21	15.02.21	1	1	1	1	**0**
Latvia	02.03.	27.03.20	27.03.20	5	1	1	1	-
Greece	26.02.	28.12.20	28.12.20	1	1	1	1	-
Czech Republic	01.03.	n/a	n/a	37	1	1	1	1
Taiwan	21.01.	12.04.20	12.04.20	6	1	1	1	**0**
Colombia	06.03.	27.03.20	27.03.20	70	1	1	1	15
Thailand	13.01.	18.03.20	20.03.20	4	1	1	1	3
Hong Kong	22.01.	n/a	n/a	8	1	1	1	1
France	24.02.	09.09.14	29.02.20	189	1	1	**0**	205
Slovenia	04.03.	15.11.20	10.03.21	2	1	**0**	1	**0**
Luxembourg	29.02.	02.04.20	23.04.20	31	1	**0**	1	5
Netherlands	27.02.	n/a	n/a	28	1	**0**	1	8
Germany	27.01.	25.03.20	25.03.20	355	**0**	1	1	-
Bulgaria	08.03.	23.07.20	24.07.20	2	1	1	**0**	**0**
Switzerland	25.02.	05.11.19	05.03.20	20	**0**	1	1	2
Sweden	31.01.	27.03.20	27.03.20	6	1	**0**	1	6
India	30.01.	07.05.20	07.05.20	n/a 411 catalogs	1	1	**0**	44
USA	20.01.	04.02.15	31.01.20	505	**0**	1	1	**0**
Philippines	30.01.	11.10.20	20.05.21	10	1	**0**	1	-

## Data Availability

Data supporting reported results are published in Zenodo open repository-https://doi.org/10.5281/zenodo.5142245, accessed on 30 July 2021.
